# Analysis of health outcomes in acute myocardial infarction: a value-based healthcare approach

**DOI:** 10.15649/cuidarte.3796

**Published:** 2024-06-21

**Authors:** Anderson Bermon, Maricel Licht-Ardila, Edgar Fabián Manrique-Hernández, Alexandra Hurtado-Ortiz

**Affiliations:** 1 Fundación Cardiovascular de Colombia, Piedecuesta, Santander, Colombia. Universidad CES, Medellín, Colombia. E-mail: andebermon@gmail.com Universidad CES Universidad CES Medellín Colombia andebermon@gmail.com; 2 Fundación Cardiovascular de Colombia, Piedecuesta, Santander, Colombia. E-mail: maricellicht@fcv.org Fundación Cardiovascular de Colombia Piedecuesta Santander Colombia maricellicht@fcv.org; 3 Fundación Cardiovascular de Colombia, Piedecuesta, Santander, Colombia. E-mail: fabianmh1993@gmail.com Fundación Cardiovascular de Colombia Piedecuesta Santander Colombia fabianmh1993@gmail.com; 4 Fundación Cardiovascular de Colombia, Piedecuesta, Santander, Colombia. E-mail: alexandrahurtado@fcv.org Fundación Cardiovascular de Colombia Piedecuesta Santander Colombia alexandrahurtado@fcv.org

**Keywords:** Acute Myocardial Infarction, Value-Based Health Care, Hospital Costs, Outcome Assessment, Infarto Agudo de Miocardio, Atención Médica Basada en Valor, Costos de Hospital, Evaluación de Resultados, Enfarto Agudo do Miocárdio, Cuidados de Saúde Baseados em Valores, Custos Hospitalares, Avaliação de Resultados

## Abstract

**Introduction:**

Understanding the impact of value-based healthcare and various healthcare payment models on the health outcomes of patients with acute myocardial infarction (AMI) is pivotal for guiding clinical strategies and decisions.

**Objective:**

To compare health outcomes and costs associated with healthcare for AMI patients under insurance prospective global payment (PGP) and fee-for-service models.

**Materials and Methods:**

A retrospective cohort study encompassing AMI patients was conducted from 2021-2023. Convenience sampling of participants over 18 years of age diagnosed with type 2 myocardial infarction was conducted. Analysis was based on Colombian healthcare system payment models: PGP and fee-for-service.

**Results:**

The study involved 2134 patients, 657 (31%) under PGP and 1477 (69%) under fee-for-service. Length of hospital stay was associated with the payment model (coefficient -0.68, CI 95%: 0.40 to 0.98, p=0.037). Payment models also correlated with costs (845 USD, CI 95%: 87.92 to 1601; p=0.02). In-hospital mortality is not associated with either of the two contracting models. Quality-adjusted life years (QALYs) totaled 1.6 over a 2-year follow-up

**Discussion:**

It is evident that throughout the care cycle at the Center of Excellence for Acute Myocardial Infarction, there is added value for patients with the PGP model, as the costs are lower and health outcomes comparable to the fee-for-service model.

**Conclusions:**

The findings of this study underscore the importance of understanding the relationship between value-based healthcare, different healthcare payment models, and health outcomes in AMI patients.

## Resumen

**Introducción:**

Es fundamental comprender cómo la atención en salud basada en el valor y los diversos modelos de contratación afectan los resultados de salud de los pacientes con infarto agudo de miocardio para orientar las estrategias y decisiones clínicas.

**Objetivo:**

Comparar los resultados de salud y los costos asociados a la atención en salud de pacientes con infarto agudo de miocardio bajo los modelos de contratación de pago global prospectivo y pago por servicio.

**Materiales y Métodos:**

Estudio de cohortes retrospectivo en pacientes con infarto agudo de miocardio realizado entre 2021 y 2023. Se realizó un muestreo por conveniencia de participantes mayores de 18 años con diagnóstico de infarto de miocardio tipo 2. El análisis se basó en dos modelos de contratación del sistema de salud colombiano: modelo de pago global prospectivo y modelo de pago por servicio.

**Resultados:**

En el estudio participaron 2134 pacientes, 657 (31%) bajo el modelo pago global prospectivo y 1477 (69%) bajo el modelo de pago por servicio. La duración de la estancia hospitalaria se asoció con el modelo de contratación (coeficiente 0,68; 95% IC: 0,04 a 1,33; p=0,037). El modelo de contratación mostró correlación con los costos (coeficiente 845; 95% IC: 87,92 a 1601; p=0,02). La mortalidad intrahospitalaria no se asocia a ninguno de los dos modelos de contratación. Los AVAC totalizaron un valor de 1,6 durante un seguimiento de 2 años.

**Discusión:**

Es evidente que en todo el ciclo de atención en el Centro de Excelencia para el Infarto Agudo de Miocardio existe un valor añadido para el paciente bajo el modelo de contratación de pago global prospectivo, ya que los costos son menores con resultados en salud comparables al modelo de contratación de pago por servicio.

**Conclusiones:**

Es crucial comprender la relación entre la atención en salud basada en el valor, los distintos modelos de contratación y los resultados de salud de pacientes con infarto agudo de miocardio.

## Resumo

**Introdução:**

Compreender o impacto dos cuidados de saúde baseados em valor e de vários modelos de contratação nos resultados de saúde em pacientes com enfarte agudo do miocárdio é fundamental para orientar estratégias e decisões clínicas.

**Objetivo:**

Comparar os resultados de saúde e os custos associados aos cuidados de saúde para pacientes com infarto agudo do miocárdio sob modelos prospectivos de pagamento global e taxa por serviço de seguros.

**Materiais e Métodos:**

Estudo de coorte retrospectivo abrangendo pacientes com Infarto Agudo do Miocárdio, realizado de 2021 a 2023. Amostra de conveniência de participantes com 18 anos ou mais, com diagnóstico de infarto do miocárdio tipo 2. A análise baseou-se nas modalidades de contratação do sistema de saúde colombiano: modelo de pagamento global prospectivo e taxa por serviço.

**Resultados:**

O estudo envolveu 2.134 pacientes, com 657 (31%) sob pagamento global prospectivo e 1477 (69%) sob taxa por serviço. O tempo de internação esteve associado ao modelo (Coeficiente 0,68, IC 95%: 0,04 a 1,33, p=0,037). Tipos de contratos correlacionados com custos (Coeficiente 845, IC 95%: 87,92 a 1601; p=0,02). A mortalidade intra-hospitalar não está associada a nenhum dos dois modelos de contratação. QALYs totalizaram 1,6 em um acompanhamento de 2 anos.

**Discussão:**

É evidente que ao longo de todo o ciclo de atendimento no Center of Excellence for Acute Myocardial Infarction, há valor agregado para o paciente com o Modelo de Pagamento Global Prospectivo, pois os custos são menores com resultados de saúde comparáveis à taxa por serviço tipo de contratação.

**Conclusões:**

Compreender a relação entre cuidados de saúde baseados em valor, diferentes modelos de contratação e resultados de saúde em pacientes com enfarte agudo do miocárdio é crucial.

## Introduction

Acute myocardial infarction (AMI) remains a significant global health challenge and is a leading cause of morbidity and mortality on a worldwide scale^1^^, ^^2^. In Colombia, the impact is particularly relevant, ranking among the top five causes of mortality during the years 2020-2021^3^. Additionally, the average cost of AMI was estimated to be 6234 USD^4^, and 1,116,284 Disability-Adjusted Life-Years (DALYs) were calculated nationwide in an 8-year study^5^. This condition is characterized by the sudden interruption of blood flow to the heart due to blockage of the coronary arteries, resulting in the death of a segment of heart tissue. Given the critical nature of AMI, timely and comprehensive patient care is imperative^6^. Immediate attention followed by high-quality, interdisciplinary management plays a pivotal role in improving patient conditions and preventing recurrent events^7^. This underscores the need for lifestyle interventions and ongoing assessments with a value-based healthcare approach to ensure holistic well-being^8^.

Value-based medicine (VBM) is an innovative paradigm that aims to optimize the relationship between clinical outcomes and healthcare resources. Unlike conventional approaches focusing solely on clinical effectiveness, VBM integrates quality, efficiency, and cost considerations into medical decision-making^9^. It is based on the premise of providing the most effective care possible, maximizing patient benefits while minimizing unnecessary costs. In a scientific context, VBM is emerging as a crucial approach to assessing the effectiveness and sustainability of medical interventions, providing a solid foundation for informed decision-making and continuous improvement in healthcare. This multidimensional approach contributes significantly to the advancement of clinical research by providing a comprehensive framework that goes beyond traditional boundaries, thereby promoting the delivery of high-quality healthcare and the efficient use of resources^10^^, ^^11^.

The Colombian healthcare system allows for various payment modalities, including the Prospective Global Payment (PGP), which is defined as a payment model for a specific group of individuals. Under PGP, a predetermined fixed amount is agreed upon in advance to provide services or supply of health technologies to that population over a defined period. The PGP model focuses on providers managing care effectively with the allocated resources and involves a moderate financial risk transfer. This approach is conducive to better adherence to protocols and health outcomes. The PGP model is similar to other prospective payment systems used around the world; it is a reimbursement method in which payment is based on a predetermined fixed amount based on a patient classification system that groups patients into resource homogeneous groups, with each hospital receiving a fixed, predetermined fee for each patient falling into a given group^12^.

Another payment method is fee-for-service (FFS), in which the system makes individual payments retroactively for each service provided without transferring financial risk to the provider. In the FFS model, the focus is typically on the volume and quantity of services provided, regardless of the ultimate outcome^13^. This article aims to compare health outcomes and costs associated with healthcare for patients covered by PGP and FFS models following AMI from 2021 to 2023.

## Materials and Methods

The information used in this retrospective cohort study was retrieved from the Center of Excellence for Acute Myocardial Infarction (CEAMI) database of the Fundación Cardiovascular de Colombia (FCV) between 2021 and 2023. Convenience sampling was conducted among all patients treated at the CEAMI who met the inclusion criteria, resulting in a total of 2134 patients included. Data were recorded within a project created on the REDCap platform^14^, and the database is available at Zenodo^15^. CEAMI is a healthcare initiative ofthe FCV, endorsed by the International Joint Commission, which certifies compliance with international standards of care and best medical practices^16^. CEAMI comprises specialists in cardiology, hemodynamics, psychology, nutrition, social work, physiotherapy, and nursing who work collaboratively to provide comprehensive care to patients in all their biopsychosocial dimensions. Moreover, it offers in-hospital education using resources such as booklets and workshops to empower patients to understand their condition and make informed treatment decisions. It also provides in-person and phone follow-up throughout the first year post event, with the goal of minimizing rehospitalizations and reinforcing educational efforts.

Patients admitted to the CEAMI and participants in this study are adults (aged 18 and older) who have experienced type-1 AMI and have been treated at the FCV. Sociodemographic and clinical variables, including comorbidities, risk scales (TIMI, Killip, and GRACE), laboratory results, details of medical procedures, and associated costs, were collected. Health outcomes were in-hospital all-cause mortality, length of hospital stay, quality of life (EQ-5D), QALYs, and readmissions (hospital admission within 30 days after discharge for AMI). For the analyses, patients were classified by payment models, such as PGP and FFS.

In the statistical analysis, categorical variables were described using percentages and absolute frequencies. For continuous variables, normality was assessed using the Shapiro-Wilk test. Variables with normal distribution were described using means and standard deviations, while those with non normal distribution were described using median and interquartile ranges (IQR). Multivariate models were constructed to examine the association between the independent variables (payment models) and each of the health outcomes. Logistic regression models were used for in-hospital mortality and readmission. Additionally, linear regression models were constructed for hospital stay and costs. QALYs were calculated using the EQ-5D quality of life scale. Statistical significance was considered at p<0.05. Stata 17 statistical software was used for the analysis.

### Ethical considerations

This study adhered to current guidelines for clinical research and received prior approval from the Research Ethics Committee of the Fundación Cardiovascular de Colombia (FCV). The guidelines established in Resolution 008430 of 1993 by the Ministry of Health ofColombia and the Declaration of Helsinki of 1964, as adapted in its latest revision of October 2013, were followed. The recommendations of the Good Clinical Practice Guidelines in Clinical Research and the fundamental ethical principles inherent in this type of research design, including the Belmont Report’s principles of respect for individuals, beneficence, and justice, were applied. Ethical principles and privacy regulations governing patient data management were strictly adhered to protect confidentiality and sensitive information. The analyzed information was anonymized and is under the custody of the FCV.

## Results

### Description of the Study Population

A total of 2134 CEAMI patients were included in the study. Of these, 31% were enrolled in the PGP model, while the remaining 69% were enrolled in the FFS payment model. The median age was 67 years (IQR 59-74.5) in the PGP group and 66 years (IQR 58-74) in the FFS group, with no statistically significant differences (P=0.26). The FFS group had a higher proportion of men (72.65% vs. 66.97%, p=0.008). Regarding marital status, most participants were married or cohabiting, with a higher proportion in the FFS payment model (66.18% vs. 59.87%, p=0.006) (Table 1).

Regarding clinical variables, non-ST-segment elevation myocardial infarction (nSTEMI) was more frequent in both groups, with no significant differences between them (59.54% vs. 58.57%, p=0.65). Additionally, a higher proportion of patients was undergoing percutaneous coronary intervention (PCI) in the PGP model (81.68% vs. 77.82%, p=0.04). Conversely, the FFS model showed a higher proportion of patients undergoing coronary artery bypass grafting (CABG) (15.88% vs. 21.78%, p=0.002). In-hospital mortality rates were 4.43% and 4.15% in the PGP and FFS groups, respectively (p=0.76). Regarding medical history, the prevalence of arterial hypertension and heart failure was higher in the FFS group. At the same time, diabetes mellitus, dyslipidemia, and chronic kidney disease were more prevalent in the PGP group, as shown in Table 1.

**Figure 1:**
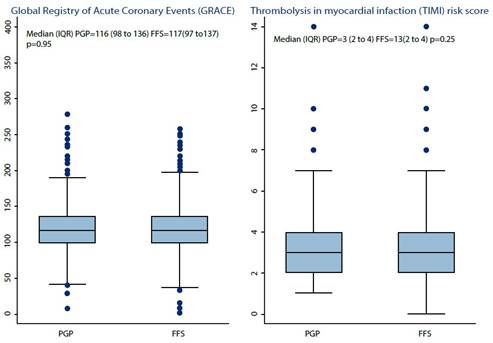
Patient’s risk scales

Patients in the FFS group had a higher body mass index (BMI) than the PGP group (median 25.7 kg/m2 vs. 25 kg/m2, p<0.001). Regarding Killip, TIMI, and GRACE scores, no statistically significant differences were observed between the two groups (Figure 1). Similarly, creatinine levels were comparable in both groups, with no statistically significant differences identified (0.91mg/dl vs. 0.93mg/dl, p=0.02). The average length of hospital stay was one day longer in the FFS group than in the PGP group (7.1 days vs. 6.1 days, p=0.004). Additionally, lifestyle habits such as smoking and alcohol consumption were reported by 12.63% and 13.93% in the PGP group and 4.57% and 5.75% in the FFS group, respectively. On the other hand, the 30-day mortality was 1.07% in PGP and 0.74% in FFS, while 30- day readmissions were 3.65% and 4.19%, respectively. Finally, the patients in the FFS group incurred higher costs than those in the PGP group (14%, p=0.0377) (Table 1).

**Table 1:** General Characteristics of the AMI Population

Variable	PGP (n=657) n(%)	FFS (n=1477) n(%)	p-value
Age. Median (IQR)	67 (75-66)	66 (58-74)	0.26
Sex			<0.01
Female	217 (33.03)	404 (27.35)	
Male	440 (66.97)	1073 (72.65)	
Marital status			<0.01
Single	250 (40.13)	490 (33.82)	
Married or cohabiting	373 (59.87)	959 (66.18)	
Risk factors	204 (91.07)	405 (89.01)	0.41
Hypertension	437 (66.51)	994 (67.21)	0.75
Diabetes	221 (33.64)	484 (32.72)	0.68
Dyslipidemia	216 (32.88)	463 (31.34)	0.47
Kidney failure	73 (11.11)	125 (8.45)	0.05
Heart failure	15 (2.28)	48 (3.25)	0.22
Alcohol consumption	30 (4.57)	85 (5.75)	0.26
Active tobacco use (1 year before admission)	83 (12.63)	206 (13.93)	0.42
Coronary heart disease	80 (12.18)	171 (11.56)	0.68
STEMI	265 (40.46)	609 (41.43)	0.67
Killip Classification			0.58
Class I	441 (77.92)	976 (80.33)	
Class II	64 (11.31)	125 (10.29)	
Class III	39 (6.89)	79 (6.50)	
Class IV	22 (3.89)	35 (2.88)	
LVEF. Median (IQR)	50 (40-55)	50 (40-55)	0.85
Troponin I (ng/ml). Median (IQR)	3016.6 (467-16,520)	3264.5 (520.8-18,736)	0.33
LDL level (mg/dl). Median (IQR)	114.56 (85-145)	113 (83- 144)	0.45
BMI. Median (IQR)	25 (23-28)	25.7 (23-28)	<0.01
Creatinine level (mg/dl). Median (IQR)	0.91 (0.76-1.16)	0.93 (0.8-1,17)	0.02
Creatinine level at 48 hours (mg/dl). Median (IQR)	0.93 (0.77-1.3)	0.98 (0.82-1.23)	0.27
PCI	535 (81.68)	1144 (77.82)	0.04
CABG	104 (15.88)	320 (21.78)	<0.01
In-hospital mortality			0.77
Yes	29 (4.43)	61 (4.15)	
No	626 (95.57)	1409 (95.85)	
Death within 30 days	7 (1.07)	11 (0.74)	0.55
Readmission	24 (3.65)	62 (4.19)	0.55
Total length of hospital stays (days). Median (IQR)	4 (2-9)	4 (2-10)	0.04
Costs (USD). Mean (SD)	6009 (779)	6981 (545)	0.04

*BMI: body mass index; CABG: coronary artery bypass grafting; FFS: fee for service; IQR: interquartile range; LDL: low-density lipoproteins; LVEF: left ventricular ejection fraction; PCI: percutaneous coronary intervention; PGP: prospective global payment; SD: standard deviation; STEMI: T-elevation myocardial infarction; USD: United States Dollar.*

### In-hospital mortality

The multivariate analysis of in-hospital mortality is detailed in Table 2. Men exhibit a lower susceptibility to death during their hospital stay, as evidenced by an adjusted odds ratio (OR) of 0.46 (CI 95%: 0.28 to 0.75, p<0.01). Conversely, patients diagnosed with ST-segment elevation myocardial infarction(STEMI) have a higher likelihood of mortality compared to patients with nSTEMI, with an OR of 1.71 (CI 95%: 1.04 to 2.8, p=0.03). Notably, there were no substantial differences in mortality outcomes based on the type of contracting, as indicated by an OR of 1.003 (CI 95%: 0.60 to 1.66, p=0.99). Similarly, it was observed that the likelihood of mortality increased with each day of stay with an OR of 1.05 (CI 95%: 1.03 to 1.08, p<0.01).

### Length of stay

Regarding the multivariate model for length of hospital stay, several variables exhibited significant associations, including Killip, PCI performance, payment model, and age (**Table 2**). Age showed a statistically significant association, indicating that for every one-year increase in age, the length of stay decreased (coefficients -0.027, CI 95%: -0.05 to 0.045, p<0.001). The payment model also showed a significant association, with patients in the FFS model having longer hospital stays than those in the PGP model (coefficients 0.68, CI 95%: 0.04 to 1.33, p=0.037). In addition, patients undergoing PCI were discharged earlier (coefficients -0.30, CI 95%: -3.37 to 2.22, p<0.001). The Killip classification, which indicates the patient’s admission severity, was associated with the length of hospital stay, showing that patients classified as Killip III (coefficient 2.83, CI 95%: 1.59 to 4.06, p<0.001) and IV (coefficient 4.25, CI 95%: 2.53 to 5.97, p< 0.001) had longer stays (3 and 4 days each) compared to patients classified as Killip I…

**Table 2:** Multivariate analysis of health and cost outcomes

Variable	In-hospital mortality		Readmission		Total length of stay		Costs	
OR	p-value	OR	p-value	coefficient	p-value	coefficient	p-value
Age	1 (0.99 to 1.01)	0.44	0.99 (0.97 to 1.01)	0.83	-0.027 (-0.05 to 0.045)	<0.01	-23.27 (-55.04 to 8.49)	0.15
Sex (Reference Female)	0.46 (0.28 to 0.75)	<0.01	-	-	0.29 (-0.40 to 0.98)	0.41	1159 (356.22 to 1961.4)	0.005
Payment model (Reference PGP)	1.003 (0.60 to 1.66)	0.99	1.23 (0.74 to 2.04)	0.41	0.68 (0.04 to 1.33)	0.037	845 (87.92 to 1601)	0.02
Marital status (Reference Single)	-	-	1.86 (1.09 to 3.19)	0.02	-	-	-	-
STEMI	1.71 (1.04 to 2.8)	0.03	-	-	-	-	-	-
BMI	0.985 (0.93 to 1.04)	0.58	-	-	-	-	-	-
Hypertension	-	-	1.69 (0.96 to 2.98)	0.06	-	-	-	-
Diabetes	-	-	1.25 (0.79 to 1.99)	0.33	-	-	-	-
Coronary heart disease	-	-	1.65 (0.93 to 2.93)	0.08	-	-	-	-
Kidney failure	-	-	1.55 (0.81 to 2.98)	0.18	-	-	-	-
Dyslipidemia	0.832 (0.5 to 1.4)	0.49	-	-	-	-	-	-
Active tobacco use (1 year before admission)	-	-	0.54 (0.23 to 1.28)	0.16	-	-	-	-
PCI	-	-	1.64 (0.87 to 3.08)	0.12	-0.30 (-3.77 to 2.22)	<0.001	80.39 (-815 to 976)	0.86
Killip II	-	-	-	-	1.69 (0.69 to 2.68)	0.001	817.02 (-340.09 to 1974.13)	0.16
Killip III	-	-	-	-	2.83 (1.59 to 4.06)	<0.001	3317 (1880.76 to 4753.16)	<0.001
Killip IV	-	-	-	-	4.25 (-2.53 to 5.97)	<0.001	11736.32 (9728.80 to 13743.83)	<0.001
Troponin (ng/ml)	1 (1 to 1)	<0.001	-	-	-	-	-	-
Death within 30 days	-	-	5.62 (1.47 to 21.38)	0.01	-	-	-	-
Total length of stay (days)	1.05 (1.03 to 1.08)	<0.001	-	-	-	-	-	-

*Note: The p-values for in-hospital mortality and readmission models were derived from a logistic regression model, whereas the p-value for hospital stay and costs was derived from a linear regression model. BMI: body mass index; PCI: percutaneous coronary intervention; STEMI: T-elevation myocardial infarction; USD: United States Dollar.

### Readmissions

The multivariate model adjusted for 30-day readmissions shows that patients who were married or cohabiting are nearly twice as likely to be readmitted to the CEAMI compared to single, widowed, or separated patients (OR 1.86, CI 95%: 1.09 to 3.19, p=0.02). Similarly, patients who died within the initial 30 days post-discharge show an increased likelihood of readmission (OR 5.62, CI 95%: 1.47 to 21.38 p=0,01). The multivariate analysis for 30-day mortality revealed noteworthy findings. Patients undergoing PCI have an almost twofold increased risk of death at 30 days compared to patients not undergoing PCI (HR 1.57, CI 95%: 1.37 to 1.79, p<0.01).

### Costs

**Figure 2:**
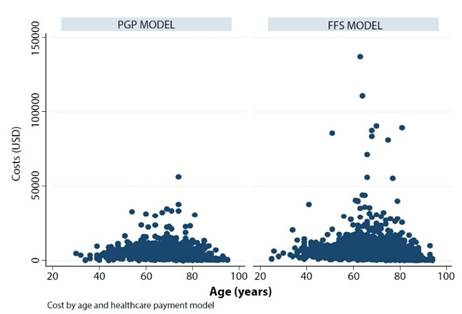
Cost by age and payment model

When examining the cost analysis results, an association with sex was observed, with men having a cost increase of 1159 USD compared to women (CI 95%: 356.22 to 1961.4, p<0.005). Similarly, when evaluating costs by payment model, it is evident that the FFS group represents a higher cost of healthcare compared to PGP (845 USD, CI 95%: 87.92 to 1601, p=0.02) (Figure 2). Likewise, it was observed that the patient’s admission severity, as determined by Killip III and IV classes, resulted in higher costs compared to Killip class I patients (3317 USD, CI 95%: 1880.76 to 4753.16, p<0.001 and 11736.32 USD (CI 95%: 9728.80 to 13743.83, p<0.001, respectively).

In this research, we calculated QALYs as a comprehensive measure of both quantity and quality of life. The results revealed that, regardless of the payment model, the total QALYs over the 2-year follow up period were 1.6 years. This suggests that, as a result of the care provided by the CEAMI, patients experience optimal health for 80% of the time assessed.

## Discussion

This analysis compared healthcare payment models in Colombia and found that the PGP model bears lower healthcare costs than the FFS model. This finding can be explained by the fact that, in clinical practice, patients in the PGP model face fewer barriers to accessing procedures, interventions, and consultations because there is no need for insurance authorization^17^. In Colombia, regulations such as Decree 4747 of 2007 govern the need for authorization to access certain services, but the PGP model has the advantage of significantly minimizing these procedures^18^. As a result, medical care becomes more efficient, resulting in fewer days of hospital stay (as demonstrated by adjusting the analyses for sex, age, payment model, type of intervention, and Killip classification)^19^^, ^^20^.

Initially, we observed that the distribution of patients by payment model (31% PGP and 69% FFS) aligns with national reports, where up to 80% of the population is generally treated using the FFS model^21^. Similarly, our study population showed that men had a higher prevalence of AMI^22^ than women in both payment models, consistent with established trends in scientific evidence^23^. Conversely, the association between marital status and a higher prevalence of the disease among married or cohabiting individuals differs from the commonly reported trend. Studies have shown that singles have a higher prevalence of the disease and present unfavorable outcomes^24^. These distribution dynamics may be related to other variables associated with lifestyle and behaviors related to regional customs; however, in this study, we did not measure variables such as diet, occupation, or physical activity.

It is worth noting that, in the descriptive analysis, the clinical variables were similarly distributed in both groups. Patient complexity, as determined by the type of infarction, and risk scales such as TIMI, GRACE, and Killip were comparable. Characteristics that showed statistically significant differences between the two groups included sex, marital status (although the majority of patients in both groups were married or cohabiting), the proportion of patients undergoing PCI and CABG, BMI, length of stay, and costs, which were adjusted for by multivariate analysis.

In the context ofthe assessed health outcomes, the multivariate analysis underscored the significance of sex in mortality, revealing that women were more susceptible to death during their hospital stay (p=0.01)^25^. This finding emphasizes the importance of considering sex-specific factors in the management of acute myocardial infarction. Additionally, patients diagnosed with STEMI had a higher likelihood of mortality (p=0.03), often due to complete occlusion of the involved coronary artery, underscoring the impact of prompt and effective intervention for this specific subgroup^26^.

In the analysis of hospital stays, it was observed that people at extreme ages, whether young people between 20 and 40 years old or older adults between 80 and 100 years old, had shorter hospital stays. This may be due to the fact that older adults are not candidates for CABG and, on the contrary, typically undergo PCI, which is a less invasive procedure. Additionally, the younger population may experience less severe AMIs. Thus, PCI showed a highly significant association with the length of stay, with a p-value <0.001. Patients undergoing PCI have an average one-day shorter hospital stay, as it is a less invasive procedure^27^^, ^^28^. The analysis of the length of stay allowed us to infer patients treated in the FFS payment model stayed one day longer than those in the PGP model, considering the decrease in requests for healthcare authorizations.

In our cost analysis, several variables were found to have a significant impact. Sex was identified as a contributing factor, with men being associated with increased care costs of approximately 1159 USD (356.22 to 1961.4) compared to women. This may be linked to the higher incidence observed in the male population^(29)^. Additionally, Killip class IV was linked to a significant mean cost variation of USD 11736 compared to Killip class I (p<0.001). This underscores the critical importance of early assessment and effective management, not only for clinical outcomes but also for economic considerations.

On the other hand, it is important to emphasize that the payment model is also associated with costs, which are 845 USD higher in the FFS payment model than in the PGP model. This phenomenon underlies the philosophy behind the PGP model, which returns money to the healthcare provider faster because payment is agreed upon before the service is rendered.

These findings highlight the influence of these variables on the prolongation of hospital stay and underscore the importance of considering these factors in the care assessment and management of AMI patients^28^. Identifying these variables associated with hospital stay provides a solid foundation for designing intervention strategies focused on improving efficiency and reducing the length of stay. This comprehensive approach reinforces our commitment to patient-centered care and effective resource management, promoting excellence in healthcare at our center.

The detailed QALYs revealed a fundamental aspect of the intervention conducted by the CEAMI. This outcome provides a holistic measure of health by considering both the quantity and quality of life experienced by patients. The results highlight 1.6 QALYs reported by patients, meaning that, thanks to the intervention implemented by the CEAMI, patients are able to live in an optimal state of health for approximately 80% of the first two years of follow-up. This finding suggests a significant impact on the quality of life of patients after an AMI episode, indicating that the care provided not only translates into increased life expectancy but also enhances the experience of life in terms of well being and perceived health.

These results support the relevance of the care strategies implemented by the CEAMI and reinforce its role in improving not only mortality but also the quality of life of patients who have suffered an AMI. Considering that in-hospital mortality is less than 5% regardless of the payment model, the literature reports a percentage ranging between 7 and 8.5%^30^. Furthermore, this comprehensive approach highlights the importance of considering outcome measures beyond simple survival, emphasizing patients' ability to lead healthy and satisfying lives after an acute cardiovascular event. Therefore, it is evident that throughout the care cycle at CEAMI, there is added value for the patients with the PGP model, as the costs are lower with health outcomes comparable to the FFS payment model. This not only benefits the center but also the patients who receive quality care with outcomes similar to international standards.

### Limitations

This study acknowledges several limitations. First, using the visual analog scale from the EQ5D-3L quality of life scale began in 2023, so conclusions regarding QALYs are specific to the evaluated period. Additionally, no cost adjustments were made between the periods analyzed. Furthermore, the study’s sample is another limitation, as it was selected for convenience rather than through randomization.

## Conclusion

The results of this analysis highlight the indispensable role of healthcare payment models, with particular emphasis on the potential advantages of the PGP model. Notably, the PGP model significantly reduces hospital days and healthcare costs while maintaining or potentially enhancing health outcomes for individuals experiencing AMI. Patients who died had more extended hospital stays, with women and patients with ST-segment-elevation myocardial infarction more likely to die during hospitalization. Additionally, married patients were associated with higher readmission rates.

These insights are particularly relevant given the current lack of robust evidence in Latin America and at the national level to assess health outcomes based on healthcare payment models. The limited existing evidence underscores the novelty and significance of this study, which adds valuable knowledge to this underexplored area. Moreover, this research advocates for a nuanced consideration of value-based models in healthcare policy and practice, especially within the specific context of acute myocardial infarction.

This has broader implications, suggesting that adopting value-based models could optimize patient care and resource utilization, providing a valuable framework for health policy decisions. This study not only enhances our understanding of the impact of payment models on health outcomes but also calls for further research and implementation of value-based approaches in healthcare, particularly in the Latin American context, where such evidence is currently scarce.
